# Author Correction: Life-cycle-coupled evolution of mitosis in close relatives of animals

**DOI:** 10.1038/s41586-024-07961-5

**Published:** 2024-08-30

**Authors:** Hiral Shah, Marine Olivetta, Chandni Bhickta, Paolo Ronchi, Monika Trupinić, Eelco C. Tromer, Iva M. Tolić, Yannick Schwab, Omaya Dudin, Gautam Dey

**Affiliations:** 1 Cell Biology and Biophysics, European Molecular Biology Laboratory (EMBL), Heidelberg, Germany; 2 Swiss Institute for Experimental Cancer Research, School of Life Sciences, Swiss Federal Institute of Technology (EPFL), Lausanne, Switzerland; 3 Electron Microscopy Core Facility, European Molecular Biology Laboratory, Heidelberg, Germany; 4 Division of Molecular Biology, Ruđer Bošković Institute (RBI), Zagreb, Croatia; 5 Cell Biochemistry, Groningen Biomolecular Sciences & Biotechnology Institute, University of Groningen, Groningen, The Netherlands

**Keywords:** Mitosis, Evolutionary developmental biology, Fluorescence imaging

Correction to: *Nature* 10.1038/s41586-024-07430-z Published online 22 May 2024

In the version of this article initially published, the Data availability section did not list the data deposit at https://www.ebi.ac.uk/biostudies/bioimages/studies/S-BIAD1306, which is now included in the HTML and PDF versions of the article.

