# Supplementary material for: Life-cycle-coupled evolution of mitosis in close relatives of animals
Source: Nature. 2024 May 22;630(8015):116–22. doi: 10.1038/s41586-024-07430-z (PMC11153136; doi:10.1038/s41586-024-07430-z)
Supplement: Supplementary file 1 — Supplementary Information [file 41586_2024_7430_MOESM1_ESM.pdf]

nature portfolio

https://doi.org/10.1038/s41586-024-07430-z

# Supplementary information

# Life-cycle-coupled evolution of mitosis in close relatives of animals

In the format provided by the authors and unedited

## Supplementary Information

The overall architecture of MTs in *C. perkinsii*'s spindle is strikingly similar to that of human somatic cells (Fig. 3c-f and Extended Data Fig. 8). However, *C. perkinsii*'s spindles are 3.5-folds shorter and 4.3-folds narrower than human retinal pigment epithelial-1 (RPE-1) (Extended Data Fig. 8), indicating a leaner structure. Additionally, we detect bundles of microtubules that end sharply near the equatorial plane in *C. perkinsii* spindles bearing analogy with kinetochore fibres (Fig. 3c-e). The interkinetochore distance was 4-folds smaller than in RPE-1 cells, suggesting a conserved ratio between spindle length and interkinetochore distance. Notably, near sister kinetochore fibres, we find a microtubule bundle resembling the bridging fibre in human cells, which consists of antiparallel microtubules laterally linking sister kinetochore fibres [68]. The tubulin signal intensity of the bridging fibre, $I_b$, was 41% of the tubulin signal intensity next to the kinetochore, represented by $I_{bk} = I_b + I_k$ (Fig. 3e). Using this fraction, we estimated that the number of microtubules in the bridging fibre ($I_b/I_k = 1/(I_{bk}/I_b\text{-}1)$) is 70% of the number of microtubules in the kinetochore fibre which lies between the known range of 30% for RPE-1 cells [27] and 82% for the HeLa cell line[40]. In agreement with the finding that *C. perkinsii* possesses the majority of metazoan kinetochore components, we found that its spindles contain well-defined microtubule bundles that resemble kinetochore fibres, which bind to and pull-on kinetochores in spindles across various organisms ranging from yeast to humans. The distance between sister kinetochore fibres in *C. perkinsii* and human spindles was 8-9% of the spindle length. This scaling of the interkinetochore distance suggests that the molecular and biophysical mechanisms governing spindle organisation and forces may be generally conserved between these two species. In human cells, bridging fibres balance the interkinetochore tension during metaphase [68-71], help in preventing and correcting incorrect attachments of kinetochores to microtubules [27,72], promote chromosome alignment at the spindle equator [73,74], and facilitate chromosome segregation during anaphase [75,76]. Exploring the function of bridging fibres in crucial aspects of mitosis in *C. perkinsii* will be an intriguing area for further investigation.

70. Polak, B., Risteski, P., Lesjak, S. & Tolić, I. M. PRC1-labeled microtubule bundles and kinetochore pairs show one-to-one association in metaphase. *EMBO Rep* **18**, 217–230 (2017).

71. Elting, M. W., Prakash, M., Udy, D. B. & Dumont, S. Mapping Load-Bearing in the Mammalian Spindle Reveals Local Kinetochore Fiber Anchorage that Provides Mechanical Isolation and Redundancy. *Curr Biol* **27**, 2112-2122.e5 (2017).

72. Matković, J., Ghosh, S., Ćosić, M., Pavin, N. & Tolić, I. M. *Kinetochore- and Chromosome-Driven Transition of Microtubules into Bundles Promotes Spindle Assembly*. http://biorxiv.org/lookup/doi/10.1101/2022.02.25.481924 (2022) doi:10.1101/2022.02.25.481924.

73. Jagrić, M., Risteski, P., Martinčić, J., Milas, A. & Tolić, I. M. Optogenetic control of PRC1 reveals its role in chromosome alignment on the spindle by overlap length-dependent forces. *Elife* **10**, e61170 (2021).

74. Risteski, P. *et al.* Length-dependent poleward flux of sister kinetochore fibers promotes chromosome alignment. *Cell Rep* **40**, 111169 (2022).

75. Vukušić, K. *et al.* Microtubule Sliding within the Bridging Fiber Pushes Kinetochore Fibers Apart to Segregate Chromosomes. *Dev Cell* **43**, 11-23.e6 (2017).

76. Jiang, Z. *et al.* Hyaluronan-Mediated Motility Receptor Governs Chromosome Segregation by Regulating Microtubules Sliding Within the Bridging Fiber. *Adv Biol (Weinh)* **5**, e2000493 (2021).
