# Supplementary material for: Life-cycle-coupled evolution of mitosis in close relatives of animals
Source: Nature. 2024 May 22;630(8015):116–22. doi: 10.1038/s41586-024-07430-z (PMC11153136; doi:10.1038/s41586-024-07430-z)
Supplement: Supplementary file 2 — Reporting Summary [file 41586_2024_7430_MOESM2_ESM.pdf]

nature portfolio

Corresponding author(s): Gautam Dey , Hiral Shah , Omaya Dudin

Last updated by author(s): Apr 4, 2024

Reporting Summary

Nature Portfolio wishes to improve the reproducibility of the work that we publish. This form provides structure for consistency and transparency in reporting. For further information on Nature Portfolio policies, see our Editorial Policies and the Editorial Policy Checklist.

Please do not complete any field with "not applicable" or n/a. Refer to the help text for what text to use if an item is not relevant to your study.
For final submission: please carefully check your responses for accuracy; you will not be able to make changes later.

Statistics

For all statistical analyses, confirm that the following items are present in the figure legend, table legend, main text, or Methods section.

n/a	Confirmed	
☐	☒	The exact sample size (*n*) for each experimental group/condition, given as a discrete number and unit of measurement
☐	☒	A statement on whether measurements were taken from distinct samples or whether the same sample was measured repeatedly
☐	☒	The statistical test(s) used AND whether they are one- or two-sided<br>*Only common tests should be described solely by name; describe more complex techniques in the Methods section.*
☒	☐	A description of all covariates tested
☒	☐	A description of any assumptions or corrections, such as tests of normality and adjustment for multiple comparisons
☐	☒	A full description of the statistical parameters including central tendency (e.g. means) or other basic estimates (e.g. regression coefficient) AND variation (e.g. standard deviation) or associated estimates of uncertainty (e.g. confidence intervals)
☐	☒	For null hypothesis testing, the test statistic (e.g. *F*, *t*, *r*) with confidence intervals, effect sizes, degrees of freedom and *P* value noted<br>*Give P values as exact values whenever suitable.*
☒	☐	For Bayesian analysis, information on the choice of priors and Markov chain Monte Carlo settings
☒	☐	For hierarchical and complex designs, identification of the appropriate level for tests and full reporting of outcomes
☒	☐	Estimates of effect sizes (e.g. Cohen's *d*, Pearson's *r*), indicating how they were calculated

Our web collection on statistics for biologists contains articles on many of the points above.

Software and code

Policy information about availability of computer code

Data collection	All data was collected on commercial microscopes running the following acquisition software: Zen (Zeiss), SerialEM and Atlas 3D.
Data analysis	Image analysis was performed in Fiji running ImageJ and Imaris (v992). Additional analyses were carried out using HMMER suite (3.3.2), MAFFT (7.490), TrimAL (1.2), IQTree (2.1.2), iTOL (v5)GraphPad 9, RStudio and PyCharmIDE. Segmentation and visualisation of EM images was done with 3DMod and Amira (version 2019.3 or 2020.1).

For manuscripts utilizing custom algorithms or software that are central to the research but not yet described in published literature, software must be made available to editors and reviewers. We strongly encourage code deposition in a community repository (e.g. GitHub). See the Nature Portfolio guidelines for submitting code & software for further information.

Data

Policy information about availability of data

All manuscripts must include a data availability statement. This statement should provide the following information, where applicable:

- Accession codes, unique identifiers, or web links for publicly available datasets
- A description of any restrictions on data availability
- For clinical datasets or third party data, please ensure that the statement adheres to our policy

Data availability: Data associated with the study is available at https://doi.org/10.6084/m9.figshare.c.6639812 and additional bulk microscopy datasets are available in s3 storage bucket: shahnature2024 accessible with

access key: rSmWsv6HGFcBOvMLFsKI
secret key: 9O64iOgCflVIy0FVVgNQvHb83SBIUBMUpVpHaCLP. Data can be accessed through minIO Client (https://min.io/docs/minio/linux/reference/minio-mc.html) or other s3 compatible services.

Research involving human participants, their data, or biological material

Policy information about studies with human participants or human data. See also policy information about sex, gender (identity/presentation), and sexual orientation and race, ethnicity and racism.

Reporting on sex and gender	NA
Reporting on race, ethnicity, or other socially relevant groupings	NA
Population characteristics	NA
Recruitment	NA
Ethics oversight	NA

Note that full information on the approval of the study protocol must also be provided in the manuscript.

Field-specific reporting

Please select the one below that is the best fit for your research. If you are not sure, read the appropriate sections before making your selection.

☒ Life sciences ☐ Behavioural & social sciences ☐ Ecological, evolutionary & environmental sciences

For a reference copy of the document with all sections, see nature.com/documents/nr-reporting-summary-flat.pdf

Life sciences study design

All studies must disclose on these points even when the disclosure is negative.

Sample size	All data in this study represented either qualitative or quantitative analyses extracted from unmodified microscopy images. For fluorescence and/or electron microscopy images minimum of 5-10 cells per condition/timepoint per species were obtained. Following initial analysis, additional analysis was collected for any condition with notably lower counts. Sample sizes were determined to enable rigorous testing of hypotheses raised by preliminary experimental data. Data acquisition was continued until further acquisition was causing no measurable difference in results.
Data exclusions	No data were excluded from the analyses.
Replication	All experiments were repeated with two or three independent biological replicates tested on different days with different source cultures, as specified in legends and methods section. A proportion of the experiments were repeated in two different labs. A proportion of the imaging assays were replicated by a postdoc in the lab, albeit at a different stage of development. All attempts at replication were successful.
Randomization	This is not applicable for our study. All microscopy images were obtained randomly from synchronized cell populations at different days and in some cases across two distinct laboratories.
Blinding	Data were analysed where possible by automated or semi-automated methods. In cases where manual filtering or correction was incorporated, same criteria were applied to all strains or treatments under investigation.

Reporting for specific materials, systems and methods

We require information from authors about some types of materials, experimental systems and methods used in many studies. Here, indicate whether each material, system or method listed is relevant to your study. If you are not sure if a list item applies to your research, read the appropriate section before selecting a response.

Materials & experimental systems

n/a	Involved in the study
☐	☒ Antibodies
☒	☐ Eukaryotic cell lines
☒	☐ Palaeontology and archaeology
☐	☒ Animals and other organisms
☒	☐ Clinical data
☒	☐ Dual use research of concern
☒	☐ Plants

Methods

n/a	Involved in the study
☒	☐ ChIP-seq
☒	☐ Flow cytometry
☒	☐ MRI-based neuroimaging

Antibodies

Antibodies used

Anti-Tub E7 antibody (DSHB), Anti-Tub (NB600-936 Novus Biologicals), AA344 and AA345 (ABCD antibodies), Anti-NPC MAb414 (Biolegend 902901), Goat anti-Mouse Secondary Antibody, Alexa Fluor 488(Thermo A-11001) , Goat anti-guinea pig Secondary Antibody, Alexa Fluor 568 (Thermo A-11075), Goat anti-rabbit Secondary Antibody, Alexa Fluor 568 (Thermo A78955).

Validation

All primary antibodies used in the study are well established in the field and frequently used for immunofluorescence of the targets. All antibodies were supported by manufacturer's validation statements for purposes utilised in the study.

Anti- Tub E7 was previously validated in various single-celled eukaryotes :
Choanoflagellates : https://www.sciencedirect.com/science/article/pii/S0960982213011275?via%3Dihub , https://journals.plos.org/plosbiology/article?id=10.1371/journal.pbio.3000226
Leishmania chagasi: https://www.sciencedirect.com/science/article/pii/S0021925820890899?via%3Dihub

Anti-Tub (NB600-936 Novus Biologicals) was cross-referenced against Anti-TubE7 in our experiments.

Anti-NPC MAb414 (Biolegend 902901) was previously validated in various single-celled eukaryotes :
Yeast : https://journals.biologists.com/jcs/article/135/24/jcs260240/286062/Ultrastructure-expansion-microscopy-reveals-the
Theleiria parasite: https://journals.asm.org/doi/10.1128/msphere.00709-19
Plasmodium berghei : https://www.ncbi.nlm.nih.gov/pmc/articles/PMC9601220/

Animals and other research organisms

Policy information about studies involving animals; ARRIVE guidelines recommended for reporting animal research, and Sex and Gender in Research

Laboratory animals

The study did not involve laboratory animals- only lab strains of the unicellular models Ichthyosporeans (Sphaeroforma sp. Crolimax fragrantissima, Amoebidium sp.) and Corralochytrium sp.

Wild animals

This study did not involve wild animals

Reporting on sex

For non-animal model organisms, reporting on sex is not relevant.

Field-collected samples

This study did not involve field-collected samples

Ethics oversight

Ethics approval is not required for research using unicellular holozoa.

Note that full information on the approval of the study protocol must also be provided in the manuscript.

This checklist template is licensed under a Creative Commons Attribution 4.0 International License, which permits use, sharing, adaptation, distribution and reproduction in any medium or format, as long as you give appropriate credit to the original author(s) and the source, provide a link to the Creative Commons license, and indicate if changes were made. The images or other third party material in this article are included in the article's Creative Commons license, unless indicated otherwise in a credit line to the material. If material is not included in the article's Creative Commons license and your intended use is not permitted by statutory regulation or exceeds the permitted use, you will need to obtain permission directly from the copyright holder. To view a copy of this license, visit http://creativecommons.org/licenses/by/4.0/
